# Pleura and lung

**DOI:** 10.1186/1470-7330-14-S1-O41

**Published:** 2014-10-09

**Authors:** Stefan Diederich

**Affiliations:** 1 Department of Diagnostic and Interventional Radiology; Marien Hospital, 40479 Düsseldorf, Germany

## 

Incidental pulmonary nodules in patients that are imaged for staging purposes or during follow-up may represent metastases, second primary tumours (lung cancer) or benign nodules.

The probability of pulmonary metastases or primary lung cancer differs depending on the number, size, size distribution and morphology of the nodules as well as the histologic type, stage, grade and other features of the known malignancy. A large proportion of pulmonary nodules in patients with known cancer are benign. Thus, an unconfounded diagnosis of pulmonary metastases has to be avoided in order not to falsely preclude potentially curative therapy of the primary tumour.

Synchronous or metachronous second primary lung cancers in a patient with a known lung cancer have to be differentiated from pulmonary satellite nodules representing either advanced tumour stages or metastatic disease as they may be amenable to curative surgery.

Pulmonary consolidation or ground glass attenuation most often represents infection and other non-malignant pathology but may occasionally be due to malignant lesions.

Pleural effusions and focal or diffuse pleural thickening from benign causes need to be differentiated from lesions representing pleural carcinomatosis which represents metastastic disease.

During this course examples of different incidental pulmonary and pleural lesions will be demonstrated and recommendations and guidelines for the management of the findings will be presented.
